# Treatment of Men with Central Hypogonadism: Alternatives for Testosterone Replacement Therapy

**DOI:** 10.3390/ijms22010021

**Published:** 2020-12-22

**Authors:** Veerle Ide, Dirk Vanderschueren, Leen Antonio

**Affiliations:** 1 Department of Endocrinology, University Hospitals Leuven, B-3000 Leuven, Belgium; veerle.ide@uzleuven.be (V.I.); dirk.vanderschueren@uzleuven.be (D.V.); 2 Clinical and Experimental Endocrinology, Department of Chronic Diseases and Metabolism, KULeuven, B-3000 Leuven, Belgium

**Keywords:** central hypogonadism, hypogonadotropic hypogonadism, functional hypogonadism, late-onset hypogonadism, gonadotropins, tamoxifen, clomiphene citrate, aromatase inhibitors

## Abstract

Central hypogonadism is a clinical condition, characterized by sexual symptoms and low serum testosterone levels, due to an impaired function of the hypothalamus or pituitary gland. Testosterone replacement therapy (TRT) is the standard treatment for hypogonadism, but it has some disadvantages. TRT is not a good option in men wishing to preserve fertility, nor in men with (a high risk of) prostate cancer, polycythemia, thrombophilia and severe cardiovascular disease. In this review, we discuss alternative treatments for central hypogonadism. If reversible causes are present, non-pharmacological interventions can be therapeutic. Gonadotropins are a good alternative to TRT when fertility is desired in the near future though they require frequent injections. Clomiphene citrate and tamoxifen seem to be a safe alternative for the treatment of functional central hypogonadism in men, as several studies reported a significant increase in testosterone levels with these drugs. However, their use is off-label and data supporting the efficacy of clomiphene citrate and tamoxifen on hypogonadal symptoms are insufficient. For this reason, clomiphene citrate and tamoxifen should not be used in routine clinical practice to treat sexual symptoms in men with central hypogonadism.

## 1. Introduction

The production of testosterone is driven by the hypothalamic-pituitary-gonadal (HPG)-axis. Hypothalamic gonadotropin releasing hormone (GnRH) stimulates the secretion of gonadotropins by the pituitary gland, namely luteinizing hormone (LH) and follicle-stimulating hormone (FSH). LH regulates the secretion of testosterone by the Leydig cells, whereas FSH supports spermatogenesis [1].

Testosterone deficiency can be asymptomatic or lead to a broad spectrum of symptoms ranging from sexual symptoms (reduced libido and morning erections, erectile dysfunction) to nonspecific symptoms, such as fatigue, depression, poor concentration, altered body composition with more body fat and decreased muscle mass, and lower bone mineral density [2,3].

Hypogonadism is a clinical condition characterized by hypogonadal signs and symptoms, together with low serum testosterone levels due to an impaired function of the HPG axis [1,4]. According to the Endocrine Society and European Academy of Andrology guidelines, only men with symptoms or signs of testosterone deficiency and repeatedly low serum testosterone concentrations on morning blood samples, taken in standardized conditions, should be diagnosed with hypogonadism [2,3].

In primary hypogonadism, the impaired androgen production is caused by a testicular problem, such as Klinefelter syndrome or testicular injury, resulting in high gonadotropin levels (hypergonadotropic hypogonadism). Central hypogonadism, on the other hand, is caused by impaired function of the hypothalamus or pituitary gland and characterized by low or inappropriately normal gonadotropin levels (hypogonadotropic hypogonadism) [3].

**Figure 1:**
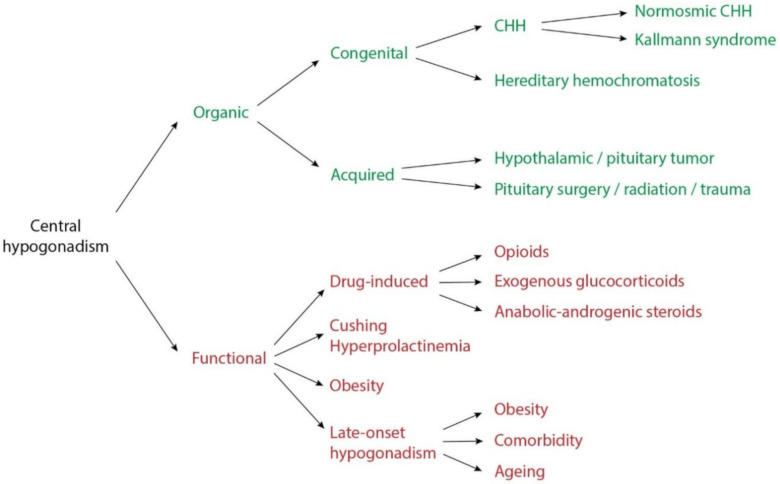
Overview of the different causes of central hypogonadism. CHH: congenital hypogonadotropic hypogonadism.

Underlying organic causes of central hypogonadism consist of congenital and acquired conditions (Figure 1). Congenital hypogonadotropic hypogonadism (CHH) is characterized by isolated central hypogonadism, due to the deficient secretion or action of GnRH. In around 50% of patients, CHH is associated with hypo- or anosmia (Kallmann syndrome), whereas in the other half, the olfactory function is preserved (normosmic CHH) [5,6,7]. Up to date, a genetic cause can be identified in almost 50% of people with CHH. Some of these genes are associated with both normosmic CHH and Kallman syndrome [7]. Hereditary hemochromatosis is an autosomal recessive disorder that disrupts the regulation of iron in the body. The iron overload can lead to organ damage and hypogonadism in later life [8]. Acquired organic central hypogonadism includes neoplasm, injury and infiltrative disorders of the hypothalamus or pituitary [1,3].

In functional central hypogonadism, the HPG axis is structurally intact, but gonadotropin production is suppressed. Frequent causes are Cushing syndrome, hyperprolactinemia, obesity and comorbidities. Drugs associated with central hypogonadism include opioids, glucocorticoids and withdrawal of anabolic-androgenic steroids [1,3,9].

Late-onset hypogonadism is a condition in ageing men that is characterized by low serum testosterone levels and sexual signs or symptoms. Testosterone levels gradually decline with age. This can become symptomatic in some men, although there is only a weak association between sexual symptoms and testosterone levels in ageing men. Therefore, the European Male Aging Study (EMAS) group suggests that only ageing men with concomitantly low total and free serum testosterone levels, and at least three sexual symptoms should be diagnosed with late-onset hypogonadism [10,11]. This clinical syndrome is associated with obesity, metabolic syndrome and chronic diseases [12,13]. However, recent evidence suggests that genes causing CHH can also predispose to mild late-onset hypogonadism [14,15].

Testosterone replacement therapy (TRT) is the standard treatment for hypogonadism. It is available in different formulations, such as transdermal patches or gels, intramuscular injections, subcutaneous pellets, nasal gels and capsules [3].

TRT has some disadvantages. It may result in gynecomastia, acne, testicular atrophy and erythrocytosis. It suppresses spermatogenesis, and thus, cannot be used in patients with a desire to have children in the near future. TRT is also contra-indicated in people with (high risk of) prostate cancer, a history of breast cancer, thrombophilia, elevated hematocrit, untreated severe obstructive sleep apnea, uncontrolled heart failure, and myocardial infarction or stroke within the last 6 months [3].

Moreover, the role of TRT to treat men with functional or late-onset hypogonadism remains controversial because of unclear indications and potential side effects [1,9]. Recently, the Testosterone Trials learned that treating men with late-onset hypogonadism with TRT resulted in a moderate improvement of sexual function, hemoglobin levels and bone mineral density, and had slightly positive effects on mood, depressive symptoms and walking distance [16].

**Figure 2:**
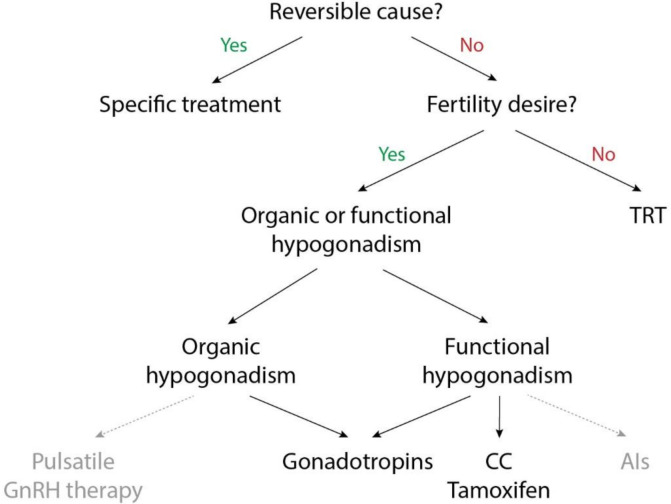
Flowchart to guide the choice of treatment for central hypogonadism. The use of clomiphene citrate, tamoxifen and AIs is off-label in male hypogonadism. The effect of these drugs on hypogonadal symptoms has not been proven. TRT: testosterone replacement therapy, GnRH: gonadotropin releasing hormone, CC: clomiphene citrate, AIs: aromatase inhibitors.

In this review, we will discuss alternative treatments for central hypogonadism (summarized in Table 1). These treatments aim to increase the endogenous testosterone production instead of administering exogenous testosterone by means of TRT [17]. The choice of treatment depends on different questions, as visualized in Figure 2.

## 2. Non-Pharmacological Treatment

As functional hypogonadism is associated with the use of certain drugs, hyperprolactinemia, obesity and chronic diseases, such as type 2 diabetes, treatment of these causes can help normalize testosterone levels [9].

The Massachusetts Male Aging Study reported that an increase in body mass index of 4 to 5 kg/m^2^ was associated with a comparable decline in total serum testosterone levels as 10 years of ageing [18]. Epidemiological data in men aged more than 50 years show that physical activity (defined as more than 32.6 metabolic equivalent hours of exercise per week) is associated with a 30% lower risk of erectile dysfunction in comparison to men who are not physically active (with less than 2.8 metabolic equivalent hours of exercise per week). Obesity increases the risk of erectile dysfunction in this population with 30% [19].

The results of the EMAS in 2736 men (mean age 58.4 years), showed that weight loss of minimal 5% of body weight in obese men resulted in a significant increase in total testosterone levels. Only a weight loss of minimal 15% had significant effects on free testosterone levels. Men who quit smoking showed a greater decrease in testosterone levels than non-smokers. Conversely, weight gain was associated with rising testosterone levels [20]. Corona et al. performed a meta-analysis of 24 studies evaluating the impact of diet and bariatric surgery on testosterone levels in obese men. Both bariatric surgery and a low-calorie diet were associated with a significant increase in testosterone levels, however the effect was more pronounced for bariatric surgery than for the low-calorie diet. The magnitude of increase in testosterone levels was associated with the amount of weight loss [21].

Heufelder et al. found that a lifestyle intervention (supervised diet and exercise) during 52 weeks significantly improved testosterone concentrations and glycemic control in 16 hypogonadal men with metabolic syndrome and newly diagnosed type 2 diabetes. The addition of testosterone gel to the lifestyle changes resulted in greater improvements of glycemic control and insulin sensitivity [22].

Several studies with a very low-calorie ketogenic diet showed a positive effect of this diet on body mass index, glycemic control and insulin resistance. These ketogenic diets mimic fasting by strongly reducing the dietary intake of carbohydrates. This leads to the synthesis of ketones which have an anorexigenic effect [23,24]. A small study in 20 patients with overweight or obesity and functional hypogonadism showed that a very low-calorie ketogenic diet during 12 weeks resulted in a mean increase in total testosterone levels of around 218% (± 53.9%) [24].

Furthermore, weight loss by lifestyle modifications is associated with improvement in sexual function, measured by the International Index of Erectile Function [25]. A meta-analysis of 12 studies in 420 patients reported that weight loss after bariatric surgery significantly improved erectile function, sexual desire and sexual intercourse satisfaction [26]. The Endocrine Society guideline emphasizes the importance of lifestyle changes in men with central hypogonadism and comorbidities [3].

Drugs that are associated with hypogonadism include opioids, glucocorticoids and estrogens [1]. Both opioids and glucocorticoids inhibit the HPG axis by suppressing GnRH secretion, resulting in decreased testosterone levels [27]. Doses of opioids and glucocorticoids should be kept as low as possible.

Hyperprolactinemia also suppresses the secretion of GnRH and the response of gonadotropins to GnRH secretion. For this reason, prolactin levels should be measured in every man diagnosed with central hypogonadism and low LH levels [3]. If hyperprolactinemia is present, one should determine the cause. In case of a pituitary prolactinoma, treatment with dopamine agonists can be indicated and can result in normalization of testosterone levels and improvement of semen quality and libido [1,3,28,29,30].

## 3. Gonadotropins

### 3.1. Mechanism of Action

In central hypogonadism, gonadotropin levels are low or inappropriately normal due to an impaired function of the hypothalamus or pituitary gland. Exogenous gonadotropins can be administered to replace endogenous gonadotropin secretion. Recombinant or urinary human chorionic gonadotropin (hCG) functions as a LH analog stimulating the production of testosterone by the Leydig cells. Human menopausal gonadotropin (hMG) is extracted from human urine and contains both FSH and LH [31]. FSH is available as a urinary derivative (highly purified FSH (hpFSH)), synthetic recombinant human FSH (rhFSH) and as corifollitropin alfa, a long-acting FSH-analog [17,31,32,33]. The urinary derivates are cheaper than the others [31]. Advantages of the recombinant forms of FSH comparing to the urinary derivatives include the limitless availability, the absence of contaminating urinary compounds, the greater stability of concentration and the greater efficacy in restoring fertility in men with hypogonadism [34]. rhFSH has a short half-life which means it has to be injected 3 times a week. Corifollitropin alfa is a FSH-analog with a similar pharmacodynamic profile as rhFSH but a longer half-life. Therefore, the frequency of corifollitropin alfa injections can be diminished to once every week [33].

### 3.2. Indications

As mentioned before, TRT cannot be used when fertility is desired in the near future. Gonadotropins, on the other hand, stimulate sperm production and can, thus, be used as part of fertility treatment in men with central hypogonadism. Current guidelines support the use of gonadotropins in men with central hypogonadism when fertility is desired [2,3,17]. In prepubertal boys, gonadotropins can be used to induce puberty with testicular growth and maturation [35,36]. Gonadotropins are, thereby, a therapeutic option in both organic and functional central hypogonadism.

### 3.3. Treatment Regimens

HCG can be administered intramuscularly or subcutaneously whereas FSH/hMG only exists as a subcutaneous formulation [1]. Doses of hCG range from 1000 to 2000 IU 2 or 3 times a week. The doses of FSH preparations usually range between 75 to 150 IU 2 or 3 times a week [5,29].

Different treatment regimens are possible. In patients with some testicular maturation (testicular volume > 4 mL), usually treatment with hCG alone is started first, followed by semen analysis after 3 to 6 months of treatment. If no sperm is detected on semen analysis at that moment, FSH or hMG is added [5,37,38]. In patients with CHH, immature testes (testicular volume < 4 mL) and no history of cryptorchidism, one study showed a benefit of pretreatment with FSH for four months (so-called FSH priming) followed by pulsatile GnRH treatment for 2 years. FSH priming induced gonadal maturation with Sertoli cell proliferation and doubled testicular volume. Furthermore, all men who received FSH pretreatment had sperm in their ejaculate [5,39,40]. Pretreatment with rFSH, followed by HCG/FSH combination therapy seems a promising treatment strategy to induce fertility in CHH patients with immature testes.

### 3.4. Results

One meta-analysis showed that gonadotropin therapy in men with hypogonadotropic hypogonadism and azoospermia induced spermatogenesis in 75% of patients [31]. In men with good testicular development, hCG monotherapy can be sufficient to induce spermatogenesis [40]. On the other hand, several studies showed that treatment with hCG alone could only induce spermatogenesis in around 50% of patients with CHH and prepubertal testes, defined by a testis volume of less than 4 mL [40,41,42]. For this reason, combined gonadotropin treatment is preferred over hCG monotherapy in patients with a lack of testicular development [5]. In one retrospective study in patients with hypogonadotropic hypogonadism, hCG treatment alone during 6 to 18 months induced a statistically significant testicular growth in 42/84 patients (50%) and spermatogenesis in 34/84 patients (40%), whereas a combination treatment of hCG and hMG led to testicular growth and spermatogenesis in respectively 56/74 patients (76%) and 48/74 patients (65%) [43]. Warne et al. performed a combined data analysis of 4 studies in 100 patients with hypogonadotropic hypogonadism treated with hCG for minimum 3 months, followed by combination therapy of hCG and rhFSH for up to 18 months. Spermatogenesis was induced in 84% of men and69% reached sperm concentrations ≥1.5 × 10^6^/mL, which is generally considered as the sperm concentration needed to achieve pregnancy [44]. As mentioned before, an alternative treatment regimen in CHH with inadequate testicular development is pretreatment with FSH monotherapy to induce testicular maturation, followed by treatment with hCG/FSH or pulsatile GnRH [39,40]. In men with prior cryptorchidism, a prepubertal testicular volume of less than 4 mL, and low serum levels of inhibin B, treatment with gonadotropins is less successful in inducing fertility [5,32,36].

Data on the impact of gonadotropin therapy on hypogonadal symptoms are limited. A small randomized placebo controlled trial showed a significant increase in body weight and lean body mass during treatment with hCG, whereas fat mass was significantly reduced [45].

### 3.5. Adverse Effects and Monitoring of Therapy

Adverse effects are limited. hCG therapy can cause gynecomastia by stimulating estrogen secretion and, less frequently, elevated hematocrit. To minimize these side effects, the dosage of hCG therapy should be kept as low as possible, aiming for testosterone levels in the low-normal range [40]. Testosterone levels should be obtained right before the subsequent injection of hCG [40]. In case of sequential or combined treatment with FSH, measuring levels of FSH is also warranted (target 4–6 IU/L) [40]. Moreover, complete blood count and PSA should be monitored. In fertility treatment, testicular volume and sperm count are a measure of response to treatment [40].

## 4. Pulsatile GnRH Therapy

Pulsatile GnRH treatment has been used to restore fertility in patients with central hypogonadism with intact pituitary function. [1]. As the physiological secretion of GnRH is episodic, continuous administration of GnRH leads to desensitization of the pituitary and suppression of gonadotropin secretion. GnRH therapy must thus be administered in a pulsatile manner by a subcutaneous pump [42,46]. Pulsatile GnRH therapy succeeds to induce spermatogenesis in 80% of men. This means the outcome of GnRH therapy and therapy with gonadotropins is similar [40]. Pulsatile GnRH therapy is expensive and requires experience and specific pump material [1,35,40] and it is no longer used in routine clinical practice.

## 5. Clomiphene Citrate and Tamoxifen

### 5.1. Mechanism of Action

Selective estrogen receptor modulators (SERMs) selectively modulate the estrogen receptor leading to variable effects in different tissues. In the central nervous system, some SERMs, namely clomiphene citrate and tamoxifen, function as antagonists to the estrogen receptor, thereby, inhibiting the negative feedback of estrogen to the hypothalamus and pituitary gland. This results in increased endogenous gonadotropin levels which stimulate testosterone production [4,17,47].

### 5.2. Off-Label Use and Treatment Regimens

Clomiphene citrate and tamoxifen are frequently used off-label in men with functional central hypogonadism [17]. In contrast to TRT, these drugs preserve fertility. However, they require a functional HPG axis and cannot be used in men with organic hypogonadism [47]. One retrospective study in 66 men identified pretreatment testicular volume ≥14 mL and mean LH level ≤6 IU/mL as predictors of good response to treatment with clomiphene citrate [48].

The advantages of these SERMs over TRT include oral administration, the maintenance of fertility, the avoidance of supraphysiological total testosterone levels and the lower risk of erythrocytosis [4,49,50].

Clomiphene citrate is usually administered as 25 or 50 mg daily or every other day. Enclomiphene citrate is a more potent, but shorter acting trans-isomer of clomiphene citrate. Dosages range from 6.25 to 25 mg/d [17]. Tamoxifen can be administered as 20–30 mg daily [51]. Other SERMs have not been tested for the treatment of hypogonadal men.

### 5.3. Results

First, it is important to notify that studies with clomiphene citrate and tamoxifen are often heterogenous and most of them lack a clear description of the study population. The majority of the studies do not distinguish between organic and functional central hypogonadism in their study population. Some even do not exclude primary hypogonadism. However, as previously mentioned, the SERMs clomiphene citrate and tamoxifen are not effective in men with primary or organic central hypogonadism. This means study results should be interpreted with caution.

Several studies reported a significant increase in testosterone levels in hypogonadal men with a steady state concentration reached 4 weeks after initiating therapy with clomiphene citrate [52,53,54,55,56].

Although, SERMs appear to have positive effects on semen parameters in men with idiopathic infertility, the effect of clomiphene citrate and tamoxifen on semen parameters in men with functional central hypogonadism remains unclear [51,57,58], as only one study reported a small increase in sperm count [4,59,60].

Concerning the impact of clomiphene citrate and tamoxifen on sexual symptoms, study results vary. A study in 86 men with hypogonadism showed a significant improvement in 5 out of 10 questions on the androgen deficiency in males (ADAM) questionnaire (including improvement in libido, life enjoyment, energy and mood level) during a mean treatment period with clomiphene citrate of 19 months. However, in 10% of patients treated with clomiphene citrate, sexual symptoms did not improve [54]. In another study in 65 men with central hypogonadism and an average age of 42 years, ADAM scoring significantly improved during treatment with clomiphene citrate [53]. A cross-sectional retrospective analysis in men treated with clomiphene citrate or TRT for symptomatic hypogonadism, reported similar improvements in ADAM scores between the group treated with clomiphene citrate and TRT [61]. In contrast, a placebo-controlled randomized controlled trial (RCT) with clomiphene citrate in 78 obese patients with functional central hypogonadism demonstrated no difference in overall ADAM questionnaire score between the 2 groups after a treatment period of only 12 weeks [62]. One should keep in mind that the ADAM score system lacks specificity. However, up to date, this is the most frequently used questionnaire to measure sexual symptoms [4]. The largest retrospective study to date included 400 patients with low testosterone levels who were treated with clomiphene citrate. In most men, an improvement in ADAM scores was observed. Of note, this is the only study that reports data on long-term use of more than three years. Also in this subgroup, 77% reported improvements in hypogonadal symptoms with clomiphene citrate [63].

Soares et al. showed significant improvements in lean mass, muscle mass and fat-free mass in the group treated with clomiphene citrate compared to placebo. Only obese patients were included in this study [62]. One study in 46 symptomatic hypogonadal patients reported a significant increase in bone mineral density during treatment with clomiphene citrate [64]. In contrast, the effect of tamoxifen on bone mineral density is unclear [64]. Data on the effects of clomiphene citrate and tamoxifen on glucose homeostasis are very limited [57].

As mentioned before, high-quality RCTs with a clear description of the study population and long-time follow-up are missing. For this reason, we consider that there is not enough evidence to justify the use of clomiphene citrate and tamoxifen as a treatment for sexual symptoms in functional central hypogonadism.

### 5.4. Adverse Effects

As clomiphene citrate and tamoxifen are used off-label to treat functional central hypogonadism, it is important to consider the side effects. Most data on adverse effects of these SERMs are derived from studies in female patients, though one cannot just extrapolate these results to the male population. However, some small studies are available that report the side effects of clomiphene citrate and tamoxifen in men. Based on data of these small studies, clomiphene citrate and tamoxifen seem to be safe for use in functional central hypogonadism [53].

Five RCTs investigated the use of tamoxifen in infertile men and reported minimal side effects [65]. Adverse effects of tamoxifen seemed to be more limited when tamoxifen was used for infertility and idiopathic gynecomastia than for male breast cancer or prostate cancer [65]. A retrospective analysis of 400 hypogonadal men, treated with clomiphene citrate for a mean duration of 25.5 months, reported side effects in only 8%. The most common adverse effects were mood changes, blurred vision, breast tenderness and weight gain. Estradiol levels were significantly increased after therapy with clomiphene citrate [63]. No side effects were reported in a study of 46 hypogonadal patients treated with clomiphene citrate for over 12 months [64]. The prevalence of secondary polycythemia is significantly lower during treatment with clomiphene citrate compared to TRT [49,50]. The risk of developing a venous thrombo-embolic event seems to increase slightly when women are treated with SERMs. However, it is unclear if this is also the case in men treated with SERMs [66]. To be safe, clomiphene citrate and tamoxifen should not be used in men with a history of venous thromboembolism (VTE).

## 6. Aromatase Inhibitors

### 6.1. Mechanism of Action and Off-Label Use

Aromatase inhibitors (AIs) inhibit the conversion of androgens to estrogens [1]. Like SERMs, AIs inhibit the negative feedback mechanism on gonadotropin secretion, leading to an increased production of testosterone [4]. Unlike the SERMs clomiphene citrate and tamoxifen, AIs reduce estrogen levels [47].

AIs are not approved to treat men with functional central hypogonadism, but anastrozole and letrozole are sometimes used as an off-label treatment [4,17].

### 6.2. Results

High quality studies with a clear definition of the study population are limited. Although testosterone levels increase in patients treated with anastrozole, no clear improvement in sexual symptoms, erectile function, body composition or muscle strength is reported [4,17,67]. A randomized comparative trial of clomiphene citrate (25 mg/d) and anastrozole (1 mg/d) in 26 infertile men with functional central hypogonadism showed significantly higher levels of testosterone in the clomiphene citrate-group than in the anastrozole-group [66].

### 6.3. Adverse Effects

Most importantly, AIs are associated with lower bone mineral density compared to TRT and placebo. This can be explained by the fact that AIs lower estrogen levels, which are important for the maintenance of bone mineral density in men [68]. Furthermore, studies with AIs in women with breast cancer show a slight increase in the incidence of venous thrombo-embolic events, although the incidence is slightly lower than in clomiphene citrate and tamoxifen [66]. In a randomized trial with clomiphene citrate and anastrozole in 26 men with central hypogonadism, one patient in the anastrozole group developed a pulmonary embolism 2 days after his final dose of anastrozole. However, this person was already at high risk for developing VTE before start of the study, judging from his history of deep vein thrombosis and inflammatory bowel disease [66]. The use of AIs in men with a history of VTE should be avoided. Other adverse effects, include hot flashes, weight gain and insomnia [47]. Prostate specific antigen levels and lipid profiles were stable under treatment with anastrozole [67].

Because of the negative effect on bone mineral density, the off-label use of AIs in functional central hypogonadism is limited.

## 7. Conclusions

Testosterone replacement therapy is considered standard therapy for male hypogonadism. However, TRT is not a good option in men wishing to preserve fertility nor in men with (high risk of) prostate cancer, polycythemia, thrombophilia and severe cardiovascular disease. Different alternative treatment options for central hypogonadism are available.

First, it is important to keep in mind that non-pharmacological interventions can be therapeutic in men with functional central hypogonadism. In particular, in obese men, lifestyle modifications are of primary importance in an attempt to restore gonadal function. Drugs negatively interfering with the HPG axis should be avoided.

Gonadotropins are a good alternative to TRT when fertility is desired in the near future though they require frequent injections. Pulsatile GnRH is a less attractive option because of the cost and the need of expertise and specific pump material.

Clomiphene citrate, tamoxifen and AIs are used off-label in men with functional central hypogonadism. Multiple studies reported a significant increase in testosterone levels with these drugs in this study population. As AIs have a negative impact on bone mineral density, their use is not recommended. Furthermore, clear clinical benefit of treatment with AIs is lacking. The SERMs clomiphene citrate and tamoxifen seem to be a safe off-label alternative for TRT for the treatment of functional central hypogonadism in men, especially in younger men who wish to maintain their fertility. However, up until now, data supporting the efficacy of clomiphene citrate and tamoxifen on hypogonadal symptoms are insufficient. Larger RCTs with a clearer definition of the study population and long-time follow-up are necessary to determine the effect of these SERMs on several outcome parameters. Until then, clomiphene citrate and tamoxifen should not be used to treat sexual symptoms in men with central hypogonadism.

## Figures and Tables

**Table 1:** Overview of the different treatment options in central hypogonadism.

Therapy	Indication	Modality	Results	Possible Adverse Effects
**TRT**	Organic and functional HG without fertility desire	IM SC Transdermal Nasal Oral	T-levels ++ Sexual function ++ Body composition ++ BMD +	Sperm production ↓ Erythrocytosis ++ PSA ↑ Gynecomastia
**Non-pharmacological**	Reversible causes	Lifestyle modification Weight loss Improved glycemic control in T2DM	T-levels + Sexual function + Body composition + BMD?	/
		Stop opioids/glucocorticoids	T-levels + Sexual function + Body composition + BMD +	Withdrawal
**Dopamine agonists**	HyperPRL	Oral	T-levels + Sexual function + Body composition / BMD +	Headache Orthostatic hypotension Nausea
**Gonadotropins**	Organic and functional HG Fertility	hCG IM/SC +/− FSH/hMG SC	T-levels ++ Spermatogenesis ++ Sexual function? Body composition? BMD?	Gynecomastia Erythrocytosis +
**Pulsatile GnRH therapy**	HG due to hypothalamic disorders	SC/IV pulsatile	T-levels ++ Spermatogenesis ++ Sexual function? Body composition? BMD?	Erythrocytosis + Expensive Requires experience and material
**Clomiphene citrate** **Tamoxifen**	Functional HG (off-label)	Oral	T-levels + Spermatogenesis + Sexual function? Body composition? BMD?	(Mood changes, blurred vision, breast tenderness, weight gain, VTE)
**Aromatase inhibitors**	Functional HG (off-label)	Oral (Anastrozole or Letrozole)	T-levels + Sexual function / Body composition / BMD−	Osteopenia Hot flashes Weight gain Insomnia (VTE)

Results: ++ strongly positive effect, + positive effect; ? effect unknown; / no effect; − negative effect.↓ decrease; ↑ increase. TRT: testosterone replacement therapy, HG: hypogonadism, IM: intramuscular, SC: subcutaneous, T: testosterone, BMD: bone mineral density, PSA: prostate specific antigen, T2DM: type 2 diabetes mellitus; hyperPRL: hyperprolactinemia, hCG: human chorionic gonadotropin, LH: luteinizing hormone, hMG: human menopausal gonadotropin, GnRH: gonadotropin releasing hormone, IV: intravenous, SERMs: selective estrogen receptor modulators, VTE: venous thromboembolism.

## Abbreviations

TRT	Testosterone replacement therapy
HPG	Hypothalamic-pituitary-gonadal
GnRH	Gonadotropin releasing hormone
LH	Luteinizing hormone
FSH	Follicle-stimulating hormone
CHH	Congenital hypogonadotropic hypogonadism
EMAS	European Male Aging Study
hCG	Human chorionic gonadotropin
hMG	Human menopausal gonadotropin
SERMs	Selective estrogen receptor modulators
ADAM	Androgen deficiency in males
RCT	Randomized controlled trial
VTE	Venous thromboembolism
AIs	Aromatase inhibitors
